# *Geodermatophilus poikilotrophi* sp. nov.: A Multitolerant Actinomycete Isolated from Dolomitic Marble

**DOI:** 10.1155/2014/914767

**Published:** 2014-07-09

**Authors:** Maria del Carmen Montero-Calasanz, Benjamin Hofner, Markus Göker, Manfred Rohde, Cathrin Spröer, Karima Hezbri, Maher Gtari, Peter Schumann, Hans-Peter Klenk

**Affiliations:** ^1^Leibniz Institute DSMZ-German Collection of Microorganisms and Cell Cultures, Inhoffenstraße 7B, 38124 Braunschweig, Germany; ^2^Instituto de Investigacióon y Formacióon Agraria y Pesquera (IFAPA), Centro Las Torres-Tomejil, Carretera Sevilla-Cazalla de la Sierra, Km 12.2, 41200 Alcalá del Río, Sevilla, Spain; ^3^Institut für Medizininformatik, Biometrie und Epidemiologie, Friedrich-Alexander-Universität Erlangen-Nürnberg, Waldstraße 6, 91054 Erlangen, Germany; ^4^Helmholtz Centre for Infection Research (HZI), Inhoffenstraße 7, 38124 Braunschweig, Germany; ^5^Laboratoire Microorganismes et Biomolécules Actives, Université de Tunis Elmanar (FST) et Université de Carthage (INSAT), 2092 Tunis, Tunisia

## Abstract

A novel Gram-reaction-positive, aerobic actinobacterium, tolerant to mitomycin C, heavy metals, metalloids, hydrogen peroxide, desiccation, and ionizing- and UV-radiation, designated G18^T^, was isolated from dolomitic marble collected from outcrops in Samara (Namibia). The growth range was 15–35°C, at pH 5.5–9.5 and in presence of 1% NaCl, forming greenish-black coloured colonies on GYM *Streptomyces* agar. Chemotaxonomic and molecular characteristics of the isolate matched those described for other representatives of the genus *Geodermatophilus*. The peptidoglycan contained *meso*-diaminopimelic acid as diagnostic diaminoacid. The main phospholipids were phosphatidylethanolamine, phosphatidylcholine, phosphatidylinositol, and small amount of diphosphatidylglycerol. MK-9(H_4_) was the dominant menaquinone and galactose was detected as diagnostic sugar. The major cellular fatty acids were branched-chain saturated acids iso-C_16:0_ and iso-C_15:0_ and the unsaturated C_17:1_ *ω*8c and C_16:1_ *ω*7c. The 16S rRNA gene showed 97.4–99.1% sequence identity with the other representatives of genus* Geodermatophilus*. Based on phenotypic results and 16S rRNA gene sequence analysis, strain G18^T^ is proposed to represent a novel species, *Geodermatophilus poikilotrophi*. Type strain is G18^T^ (= DSM 44209^T^ = CCUG 63018^T^). The INSDC accession number is HF970583. The novel R software package lethal was used to compute the lethal doses with confidence intervals resulting from tolerance experiments.

## 1. Introduction

The family cursive was originally proposed by Normand et al. [1], but a formal description of the family name was only published a decade later [2]. At the time of writing, the family comprises the genera* Blastococcus, Modestobacter,* and* Geodermatophilus* (as the type genus).* Geodermatophilus* was proposed by Luedemann [3] and was included in the Approved Lists of Bacterial Names [4]. This genus was poorly studied for a long time due to difficulties in culturing isolates [5], in spite of the fact that its members are frequently isolated from arid soils [5] and occasionally from arid and semiarid rock substrates such as rock vanish and marble [6, 7], where a variety of environmental changing factors influence their settlement, growth, and development [8]. Some of them were also isolated from rhizosphere soil [9, 10]. To enable the survival in such extreme ecological niches, where bacterial cells are suppressed to reactive oxygen species (ROS) generating-stresses, those should exhibit a very broad range of tolerance to multiple and fluctuating environmental stresses, such as solar radiation, desiccation and rehydration, temperature fluctuations, salts, and metals [8, 11], and a probable ionizing-radiation (IR) resistance. The origin of this last capability cannot be explained as adaptation to environment, suggesting an “incidental” result of tolerance to desiccation, whose DNA damage pattern is similar to that generated by ionizing radiation in* Deinococcus *species [12]. Furthermore, tolerance to hydrogen peroxide and mitomycin C as indicators of the presence of an efficient microbial oxidative stress repair and double-strand break repair system, characteristics also attributed to radiation resistance, have been widely studied [13, 14]. Multiple-stress tolerance of the type strain* Geodermatophilus obscurus* was already described by Gtari et al. [11], suggesting a correlation between tolerance profiles to desiccation, mitomycin C, hydrogen peroxide, and ionizing- and UV-radiation. Previous works of Rainey et al. [15] and Giongo et al. [16] already revealed the prevalence of IR resistant* Geodermatophilus* isolates from arid soil sample at comparatively the same radiation levels as observed for* Deinococcus* species and the predominance of species belongs to the family* Geodermatophilaceae* detected from intercontinental dust, illustrating, therefore, to resist radiation and desiccation stresses during travel in the high atmosphere.

Fourteen named species have been classified in the genus* Geodermatophilus *(ordered by the dates of effective publication of the names):* G. obscurus* [3],* G. ruber* [9],* G. nigrescens* [17],* G. arenarius* [18],* G. siccatus* [19, 20],* G. saharensis* [20, 21],* G. tzadiensis *[22, 23],* G. telluris* [24],* G. soli* and* G. terrae* [10],* G. africanus* [5, 23],* G. normandii* [25],* G. taihuensis* [26], and* G. amargosae* [27, 28]. Until now, only the genome of the type strain of the type species,* G. obscurus *G-20^T^, has been sequenced [29]. Moreover, three subspecies have been identified and named, but their names were not validly published yet: “*G. obscurus* subsp.* utahensis,*” “*G. obscurus* subsp.* dictyosporus*” [3], and “*G. obscurus *subsp.* everesti*” [30, 31]. This study describes the taxonomic position of a novel species into the genus* Geodermatophilus* based on a polyphasic approach and its tolerance to different environmental stresses.

## 2. Materials and Methods

### 2.1. Isolation

During screening for microorganisms from dolomitic marble outcrops in an agriculture area at 1150 masl in Samara, near to Namib desert (Namibia), a greenish-black strain designated as G18^T^ was isolated (in 1993) and purified as described by Eppard et al. [7].

### 2.2. Morphological and Biochemical Characterization

Cultural characteristics were tested on GYM* Streptomyces* medium (DSMZ medium 65), TSB agar (DSMZ medium 535), GPHF medium (DSMZ medium 553), R2A medium (DSMZ medium 830), GEO medium (DSMZ medium 714), PYGV medium (DSMZ medium 621), and Luedemann medium (DSMZ medium 877) for 15 days at 28°C. To determine its morphological characteristics, strain G18^T^ was cultivated on GYM* Streptomyces* medium at 28°C. Colony features were observed at 4 and 15 days under a binocular microscope according to Pelczar Jr. [32]. Exponentially growing bacterial cultures were observed with an optical microscope (Zeiss AxioScope A1) with a 100-fold magnification and phase-contrast illumination. Micrographs of bacterial cells grown on GYM* Streptomyces* broth after 7 days were taken with a field-emission scanning electron microscope (FE-SEM Merlin, Zeiss, Germany). Gram reaction was performed using the KOH test described by Gregersen [33]. Cell motility was observed on modified ISP2 [34] swarming agar (0.3%, w/v) at pH 7.2 supplemented with (l^−1^) 4.0 g dextrin, 4.0 g yeast extract, and 10.0 g malt extract. Oxidase activity was analysed using filter-paper disks (Sartorius grade 388) impregnated with 1% solution of* N,N,N*′*,N*′-tetramethyl-*p*-phenylenediamine (Sigma-Aldrich); a positive test was defined by the development of a blue-purple colour after applying biomass to the filter paper. Catalase activity was determined based on formation of bubbles following the addition of 1 drop of 3% H_2_O_2_. Growth rates were determined on plates of GYM* Streptomyces* medium for temperatures from 10 to 50°C at 5°C increments and for pH values from 4.0 to 12.5 (in increments of 0.5 pH units) on modified ISP2 medium by adding NaOH or HCl, respectively, since the use of a buffer system inhibited growth of the strains. The utilization of carbon compounds and acid production were tested at 28°C using API 20 NE strips (bioMérieux) and GEN III Microplates in an Omnilog device (BIOLOG Inc., Hayward, CA, USA) in comparison with the reference strains* G. africanus* DSM 45422^T^,* G. amargosae* DSM 46136^T^,* G. arenarius *DSM 45418^T^,* G. nigrescens *DSM 45408^T^,* G. normandii* DSM 45417^T^,* G. obscurus *DSM 43160^T^,* G. ruber* DSM 45317^T^,* G. saharensis* DSM 45423^T^,* G. siccatus* DSM 45419^T^,* G. soli *DSM 45843^T^ *, G. taihuensis *DSM 45962^T^,* G. telluris* DSM 45421^T^,* G. terrae* DSM 45844^T^, and* G. tzadiensis* DSM 45416^T^ in parallel assays. The GEN III Microplates were inoculated with cells suspended in a viscous inoculating fluid (IF C) provided by the manufacturer at a cell density of 70% transmittance (T) for* G. amargosae *DSM 46136^T^, at 75–79% T for* G. africanus *DSM 45422^T^, at 90% T for* G. arenarius* DSM 45418^T^ and* G. taihuensis *DSM 45962^T^, and at 80–83% T for all other reference strains. Respiration rates (and growth) were measured yielding a total running time of 5 or 10 days, depending on the strain, in phenotype microarray mode. Each strain was studied in two independent repetitions. Data were exported and analysed using the  opm  package for R [35, 36] v.1.0.6. Reactions with a distinct behaviour between the two repetitions were regarded as ambiguous. Clustering analyses of the phenotypic microarrays were constructed using the  pvclust  package for R v.1.2.2. [37]. Enzymatic activities were tested using API ZYM galleries according to the instructions of the manufacturer (bioMérieux).* Chemotaxonomic procedures*. Whole-cell sugars were prepared according to Lechevalier and Lechevalier [38], followed by thin layer chromatography (TLC) analysis [39]. Polar lipids were extracted, separated by two-dimensional TLC, and identified according to procedures outlined by Minnikin et al. [40] with modifications proposed by Kroppenstedt and Goodfellow [41]. Additionally, choline-containing lipids were detected by spraying with Dragendorff's reagent (Merck) [42]. Menaquinones (MK) were extracted from freeze-dried cell material using methanol as described by Collins et al. [43] and analysed by high-performance liquid chromatography (HPLC) [44]. The extraction and analysis of cellular fatty acids were carried out in two independent repetitions from biomass grown on GYM agar plates held at 28°C for 4 days and harvested always from the same sector (the last quadrant streak). Analysis was conducted using the microbial identification system (MIDI) Sherlock Version 4.5 (method TSBA40, ACTIN6 database) as described by Sasser [45]. The annotation of the fatty acids in the ACTIN6 peak naming table is consistent with IUPAC nomenclature (i.e., double bond positions identified with reference to the carboxyl group of the fatty acid), but for consistency with other publications this has been altered to numbering from the aliphatic end of the molecule (i.e., 16 : 1 CIS 9 become 16 : 1*ω*7c, etc.). The composition of peptidoglycan hydrolysates (6 N HCl, 100°C for 16 h) was examined by TLC as described by Schleifer and Kandler [46]. All chemotaxonomical analyses were conducted under standardized conditions with strain G18^T^ and cultures of the same set of reference strains as listed above for morphological and biochemical characterisations.

### 2.3. Genetic and Phylogenetic Analysis

G + C content of chromosomal DNA of strain G18^T^ was determined by HPLC according to Mesbah et al. [47]. Genomic DNA extraction, PCR-mediated amplification of the 16S rRNA gene, and purification of the PCR product were carried out as described by Rainey et al. [48]. Phylogenetic analysis was based on an alignment of 16S rRNA gene sequences from type strains of all species with effectively published names in the* Geodermatophilaceae* inferred as described by Montero-Calasanz et al. [5]. Pairwise similarities were calculated as recommended by Meier-Kolthoff et al. [49]. For DNA-DNA hybridization tests, cells were disrupted by using a Constant Systems TS 0.75 KW (IUL Instruments, Germany). DNA in the crude lysate was purified by chromatography on hydroxyapatite as described by Cashion et al. [50]. DNA-DNA hybridization was carried out as described by De Ley et al. [51] under consideration of the modifications described by Huss et al. [52] using a model Cary 100 Bio UV/VIS-spectrophotometer equipped with a Peltier-thermostatted 6 × 6 multicell changer and a temperature controller with* in situ *temperature probe (Varian).

### 2.4. Tolerance Experiments

The tolerance of strain G18^T^ and* G. obscurus* G-20^T^ (DSM 43160), as a positive control [11], to ionizing- and UV-radiation, mitomycin C, hydrogen peroxide, desiccation, and heavy metals/metalloids, was assayed using nonsporulating cultures obtained by growth in TYB medium [53] at 28°C for 5 days, washed twice with 0.9% NaCl, homogenized, and subsequently resuspended in saline solution. Ionizing-radiation experiments were carried out according to a protocol outlined by Gtari et al. [11]. To test the resistance to UV-radiation, 0.5 mL aliquots of culture suspensions was spread onto GYM* Streptomyces* agar plates in duplicate in two independent experiments and then exposed to a dose of 5–10 J*·*s^−1^ m^−2^ in a laminar flow hood equipped with crossbeam 254 nm UV sources in both side walls (Safe 2020, Thermo Scientific) for 1, 10, 30, 60, 120, 240, and 600 min. After 2 weeks at 28°C, the survival fractions were calculated based on the c.f.u. mL^−1^. The UV shadow zone was avoided. The tolerance to DNA damaging agent mitomycin C was tested in two independent experiments by incubation of cell suspension at room temperature with the antibiotic at a final concentration of 5 *μ*g*·*mL^−1^. After 1, 5, 10, 20, 40, 60, and 120 min, samples were centrifuged at 3500 rpm for 4 min, washed twice in 0.9% NaCl, and, subsequently, serially diluted. Aliquots were spread on GYM* Streptomyces* agar in duplicate. After incubation, the survival fractions were calculated based on the c.f.u. mL^−1^. To test the resistance to hydrogen peroxide, equal volumes of cell suspensions and 0.5% hydrogen peroxide were incubated at room temperature in two independent experiments. After 1, 5, 10, 20, 40, 60, and 120 min, samples were handled as was previously described in mitomycin experiments to calculate the survival fractions. For desiccation tolerance, 25 *μ*L of cell suspension were transferred to individual wells of microtiter plates in triplicate. Unsealed microtiter plates were placed in a desiccator (23.5% relative humidity) containing silica gel rubin (Fluka) at room temperature. After 20, 40, 60, 80, and 100 days, 250 *μ*L of sterile water was added to individual wells to rehydrate the desiccated cells and then incubated at room temperature for 1 hour and plated on GYM* Streptomyces* agar. The determination of survival fractions was conducted as described above. The sensitivity of strain G18^T^ to heavy metals and metalloids was determined by a growth inhibition plate assay as described by Richards et al. [54]. AgNO_3_, CuCl_2_, CoCl_2_, NiCl_2_, K_2_CrO_4_, Pb(NO_3_)_2_, and Na_2_HAsO_4_ were added to GYM* Streptomyces* medium at 0.1, 0.3, 0.5, 1.0, 2.0, 4.0, 8.0, 10.0, 30.0, and 50.0 mM. Growth was evaluated after 1 month at 28°C, determining minimum inhibitory concentration (MIC).

### 2.5. Statistical Analysis of Tolerance Experiments

To evaluate the tolerance of strain G18^T^ and* G. obscurus* DSM 43160^T^ with respect to the various physiological challenges, the median lethal dose (LD50) and the lethal dose 10 (LD10) values were computed for both strains. As the number of bacteria initially used in each experiment cannot directly be obtained and consequently, death rates or survival rates cannot be directly computed; standard models based on logistic regression models to obtain LD values are thus not available. A negative binomial model for count data [55] was used to estimate of number of survivors dependent on dose, strain, and experiment. Penalized splines [56], one for each strain, were used to allow the dose to have a nonlinear influence on survival fractions. The estimation process was stabilised by using of a square root transformation on dose. LD50 and LD10 values were subsequently estimated from the model and 95% confidence intervals were obtained using a parametric bootstrap approach [57, Chapter 5.4]. Details on model fitting and the estimation of the confidence intervals as well as code to derive LD values from survival count data with one or two strains can be found in the supplementary material (see Figure S4 in Supplementary Material available online at http://dx.doi.org/10.1155/2014/914767). All computations were done with R [58] using the R software packages  mgcv  [57] and  lethal  [59].

## 3. Results and Discussion

### 3.1. Morphological and Biochemical Characteristics

**Figure 1:**
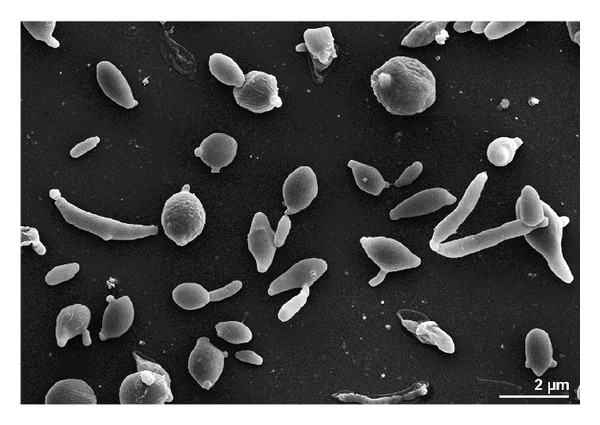
Scanning electron micrograph of strain G18^T^ grown on GYM* Streptomyces* medium for 7 days at 28°C.

Cells of strain G18^T^ were pleiomorphic and Gram-reaction-positive. Individual cells and aggregates were observed, confirming reports by Ishiguro and Wolfe [53] of synchronous morphogenesis on unspecific media and previous observations on other representatives of the genus* Geodermatophilus* [27]. In line with the original description by Luedemann [3], circular or elliptical motile zoospores and septated filaments from zoospore germination were observed (Figure 1). Young colonies were light-red in colour and turned greenish-black at maturity. Similar colours conversions were already observed by Nie et al. [17] and Montero-Calasanz et al. [18, 19, 21, 22, 25] for type strains of other representatives of the genus, such as* G. nigrescens*,* G. arenarius, G. siccatus, G. saharensis, G. tzadiensis, *and* G. normandii*, when cultivated under the same growth conditions (Table 1). Colonies were convex, nearly circular and opaque with a moist surface and an entire margin. Strain G18^T^ grew well on GYM* Streptomyces* and GEO media but did not grow on TSA, R2A, GPHF, PYGV, and Luedemann media. It grew best at 25–30°C but did not grow below 15°C or above 35°C. Growth was observed in presence of 1% NaCl and between pH 5.5–9.5 (optimal range pH 7.0–9.5). Results from phenotype microarray analysis are shown as a heatmap in the supplementary material (Figure S1) in comparison to the reference type strains of the genus* Geodermatophilus. *A summary of selected differential phenotypic characteristics is presented in Table 1. In the phenotypic clustering significant support (>95%) is obtained for* G. poikilotrophi* DSM 44209^T^,* G. nigrescens* DSM 45408^T^ and* G. normandii* DSM 45417^T^ being most similar to each other regarding the characters present in GEN III Microplates (Suppl. Figure S2).

### 3.2. Chemotaxonomic Characteristics

Analysis of cell wall components revealed the presence of* DL*-diaminopimelic acid (cell wall type III), which is consistent with other species of the genus* Geodermatophilus* [27, 38]. Strain G18^T^ displayed primarily menaquinone MK-9(H_4_) (82.5%), in agreement with values reported for the family* Geodermatophilaceae* [2], but also MK-9(H_0_) (8.8%) and MK-9(H_2_) (4.8%). Major fatty acids were iso-C_16:0_ (24.5 ± 0.2%), iso-C_15:0_ (16.6 ± 1.3%), C_17:1_ *ω*8c (13.9 ± 0.1%) and C_16:1_ *ω*7c (8.3 ± 0.1%), complemented by iso-C_16:1_ H (5.6 ± 0.9%), anteiso-C_15:0_ (4.1 ± 0.4%), anteiso-C_17:0_ (4.4 ± 0.2%), C_18:1_ *ω*9c (3.6 ± 0.1%) and C_16:0_ (2.4 ± 0.9%). The phospholipids pattern consisted of phosphatidylethanolamine (PE), phosphatidylcholine (PC), phosphatidylinositol (PI), and small amount of diphosphatidylglycerol (DPG) in accordance with profiles obtained for representatives of other* Geodermatophilus* species investigated in this study (Table 1). Phosphatidylglycerol was not detectable (see Supplementary Figure S3). This fact was already predictable based on phospholipids profiles displayed for other representatives of the genus such as* G. arenarius, G. siccatus, G. tzadiensis, G. normandii, *or* G. amargosae*, whose phosphatidylglycerol amounts were nearly imperceptible. Whole-cell sugar analysis revealed galactose as the diagnostic sugar [38] but also glucose and ribose. Genomic G + C content was 74.4 mol%.

### 3.3. Molecular Analysis

**Figure 2:**
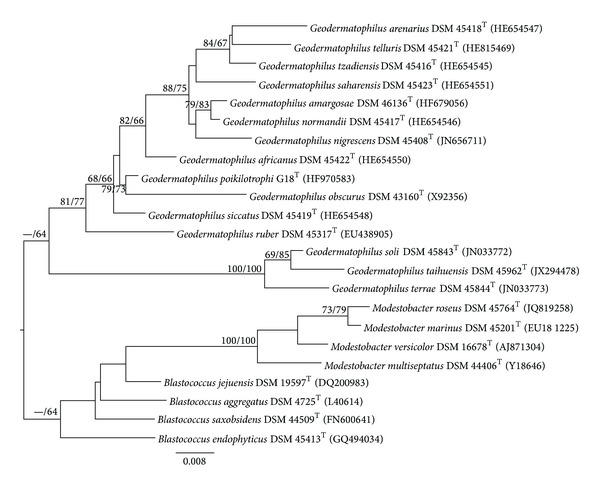
Maximum likelihood phylogenetic tree inferred from 16S rRNA gene sequences, showing the phylogenetic position of strain G18^T^ relative to the type strains within the family cursive. The branches are scaled in terms of the expected number of substitutions per site (see size bar). Support values from maximum-likelihood (left) and maximum-parsimony (right) bootstrapping are shown above the branches if equal to or larger than 60%.

The almost complete (1514 bp) 16S rRNA gene sequence of strain G18^T^ was determined. The 16S rRNA sequence showed the highest degree of similarity with the type strains of* G. siccatus* (99.1%),* G. africanus *(99.0%)*, G. amargosae* (98.5%),* G. normandii* (98.4%),* G. obscurus *(98.3%),* G. tzadiensis* (98.2%),* G. nigrescens *(98.1%),* G. ruber* (98.0%), and* G. arenarius* (98.0%). All listed closely related type strains were placed within the same phylogenetic group by both, maximum likelihood and maximum-parsimony estimations (Figure 2). The 16S rRNA gene sequences analysis thus strongly supports the assignment of strain G18^T^ to the genus* Geodermatophilus. *However, similarities in 16S rRNA gene sequence between G18^T^ and some closely related type strains indicated the need to prove the genomic distinctness of the type strain representing the novel species by DNA-DNA hybridization. Strain G18^T^ displayed a DNA-DNA relatedness of 35.3 ± 1.0% with the type strain of* G. siccatus *and 28.1 ± 2.1% with* G. africanus.* DNA-DNA hybridizations of strain G18^T^ with the type strains of* G. amargosae*,* G. normandii*,* G. obscurus*,* G. tzadiensis*,* G. nigrescens*,* G. ruber,* and* G. arenarius* were not conducted, according to Meier-Kolthoff et al. [49] that statistically confirmed that the threshold value previously established at 97% 16S rRNA gene sequence similarity was too conservative in microbial species discrimination and determined a* Actinobacteria*-specific 16S rRNA threshold at 99.0% with a maximun probability of error of 1.00% to get DNA-DNA hybridization values above the 70% threshold recommended by Wayne et al. [60] to confirm the species status of novel strains.

### 3.4. Tolerance

**Figure 3:**
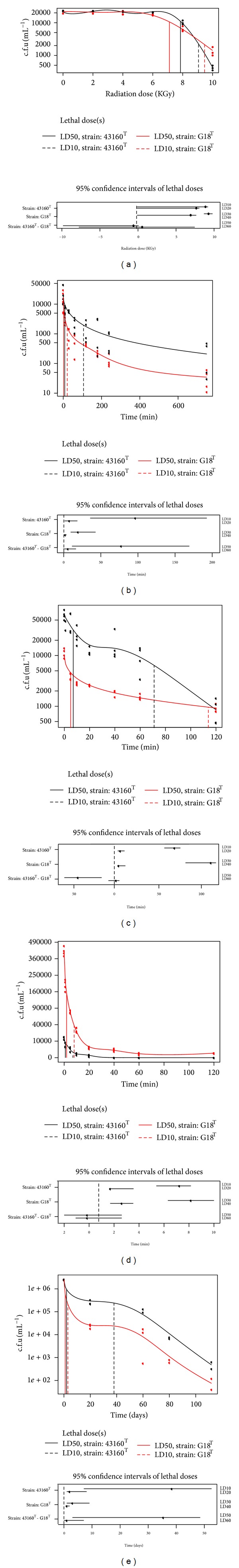
Estimation of survival following exposure to gamma-radiation (a), UV-radiation (b), mitomycin C (c), hydrogen peroxide (d), and desiccation (e) for strain G18^T^ and* G. obscurus* DSM 43160^T^ as positive control. The mean c.f.u.mL^−1^ per strain is given together with the LD50 and LD10 values in the upper panel of each figure; *y*-axis is on a logarithmic scale ((a)–(c), (e)), or on a square root scale (d). The lower panel depicts LD10 and LD50 values per strain and the differences between strains together with confidence intervals. Confidence intervals that do not contain zero (dashed vertical line) indicate significant differences to zero; in case of strain differences this indicates significant differences between strains.

Gamma-radiation survival of strain G18^T^ (Figure 3(a)) showed not significantly different inactivation kinetic as for* G. obscurus* DSM 43160^T^, which is considered as highly resistant, according to data reported by Gtari et al. [11]. Strain G18^T^ strains exhibited a shoulder of resistance similar to* D. radiodurans* R1 to approximately 5 KGy [61], but comparatively lower than the observed one by* G. obscurus* DSM 43160^T^. Nevertheless, LD10 of both, G18^T^ and* G. obscurus* DSM 43160^T^, was around 9 KGy, a dose comparatively higher than the displayed one for the high radiation resistant strain* D. radiodurans* R1 [61], although other authors reported a LD10 around 10 KGy by using the same strain [62]. UV-radiation survival curves revealed a similar progressive loss of viability in both strains during the first 10 min of exposure until levels below 50%. However, the differences between the two resistant phenotypes increased along the curve, observing a significant variation on viability at 10% survival (Figure 3(b)). According to radiated doses, strain G18^T^ and* G. obscurus* DSM 43160^T^ were capable to support the lethal effects of 6300–12600 J*·*s^−1^ m^2^ and 63600–31800 J*·*s^−1^ m^2^, respectively, sustaining a survival rate higher than 10%. Battista [63] and Shukla et al. [62] reported LD10 values of 700–1000 J*·*s^−1^ m^2^ for the highly resistant* D. radiodurans *R1. The tolerance to UV-radiation in the genus* Geodermatophilus* was already observed, in addition to* G. obscurus *DSM 43160^T^, in* G. tzadiensis* DSM 45416^T^ by Montero-Calasanz et al. [22]. Cultures of strain G18^T^ tolerated an exposure to mitomycin of nearly 120 min showing a viability rate of 10%, a value significantly higher than the one observed for the positive control (LD10 = 71 min) (Figure 3(c)). Tolerance of strain G18^T^ (LD10 = 7 min) in comparison with the positive control* G. obscurus *DSM 43160^T^ (LD10 = 8 min) to 0.5% hydrogen peroxide along the curves did not show any significant differences (Figure 3(d)). Based on desiccation survival curves given in Figure 3(e), both strains initially exhibited a similar resistance (LD50). At the first sample point (20 days), strain G18^T^ showed a survival of less than 10%, a value comparatively different to the results observed by* G. obscurus *DSM 43160^T^, whose LD10 is reached after 38 days. However, it is worth mentioning that after 110 days a remaining bacterial population of strain G18^T^ was still observed. Strain G18^T^ demonstrated thus a high tolerance to ROS-generating stresses gamma- and UV-radiation, mitomycin C, hydrogen peroxide, and desiccation comparable to the positive control* G. obscurus* DSM 43160^T^ and, in general terms, to DNA damaging-resistant* D. radiodurans* R1. This correlative tolerance between ROS-generating stresses was already widely described [11, 62] and support the hypothesis of efficient and common cellular DNA repair mechanisms. Strain G18^T^ showed the highest tolerance to AsO_4_^3−^ (MIC = 8.0 mM) followed by Pb^2+^ (MIC = 4.0 mM), CrO_4_^2−^ (MIC = 4.0 mM) and Ag^1+^ (MIC = 1.0 mM). Whereas the growth of* G. obscurus* DSM 43160^T^ was mainly inhibited by concentrations below 1.0 mM, except AsO_4_^3−^ whose sensitivity was 10 times higher (MIC = 80.0 mM) than the one observed for strain G18^T^ (Table 2). It has been widely described that the heavy metals/metalloids exposure also produces ROS generation [64]. In this study, a correspondence with other ROS-generating stresses was not observed, in agreement with data reported by Gtari et al. [11] for* G. obscurus *DSM 43160^T^, but also for* Modestobacter multiseptatus *BC501 and* Blastococcus saxobsidens *DD2, suggesting the presence of alternative mechanisms to counteract the heavy metals/metalloids stress, such as transport outside the cells [65], adsorption on exocellular structures such as melanin [66], or enzymatic reduction to less toxic forms [67, 68]. Although it is noteworthy that toxicity levels of lead and copper in* G. obscurus* DSM 43160^T^ by comparison with the results displayed by Gtari et al. [11] were much different from each other. These divergences in the levels of tolerance might be due to the differences in the media compositions [69]. In addition, it was confirmed that neither phosphate buffer nor carbon source concentration present in GYM* Streptomyces* medium caused an overestimated metals tolerance of strains, justified by the different tolerance range found in both strains and its mostly correlation with the results described by Gtari et al. [11].

Apart from the phylogenetic analysis based on 16S rRNA gene sequences, several phenotypic features support the distinctiveness of strain G18^T^ from representatives of all other* Geodermatophilus* species (Table 1). Based on the phenotypic and genotypic data presented, we propose that strain G18^T^ represents a novel species within the genus* Geodermatophilus*, with the name* Geodermatophilus poikilotrophi *sp. nov.

*Description of Geodermatophilus poikilotrophi sp. nov*..* Geodermatophilus poikilotrophi* (poi.kil.o.troph'i N. L. fem. gen. n.* poikilotrophi* referring to a bacterium that can tolerate diverse environmental stresses). 

Colonies are greenish-black-coloured, circular, and convex with a moist surface. Cells are Gram-reaction-positive, catalase positive, and oxidase negative. No diffusible pigments are produced on any of the tested media. Utilizes dextrin, D-maltose, D-trehalose, D-cellobiose, sucrose, stachyose, D-glucose, D-mannose, D-fructose, D-galactose, L-rhamnose, D-sorbitol, D-mannnitol, myo-inositol, glycerol, L-arginine, pectin, D-gluconic acid, quinic acid, methyl pyruvate, D-lactic acid methyl ester, *α*-ketoglutaric acid, D-malic acid, bromosuccinic acid, potassium tellurite, *ϒ*-amino-N-butyric acid, acetoacetic acid, propionic acid, acetic acid, as sole carbon source for energy and growth, but not turanose, D-raffinose, D-melibiose, *β*-methyl-D-glucoside, D-salicin,* N*-acetyl-D-glucosamine,* N*-acetyl-D-galactosamine,* N*-acetylneuraminic acid, 3-*O*-methyl-D-glucose, D-fucose, inosine, sodium lactate, D- and L-serine, D-arabitol, D-glucose-6-phosphate, D-aspartic acid, glycyl-L-proline, L-alanine, L-glutamic acid, L-histidine, L-pyroglutamic acid, L-galactonic acid-*γ*-lactone, glucuronamide, mucic acid, D-saccharic acid,* p*-hydroxyphenylacetic acid, citric acid, *γ*-amino-n-butyric acid, and butyric acid. Acid is produced from L-arginine and *ϒ*-amino-N-butyric acid and can be used as a sole nitrogen source but not* N*-acetyl-D-glucosamine,* N*-acetyl-D-galactosamine,* N*-acetyl-neuraminic acid, D- and L-serine, D-aspartic acid, glycyl-L-proline, L-alanine, L-histidine, L-glutamic acid, L-histidine, L-pyroglutamic acid, glucuronamide, and *γ*-amino-n-butyric acid. Positive for aesculin degradation. Negative for reduction of nitrate, denitrification, indole production and gelatin degradation. Tests for alkaline phosphatase, esterase lipase (C8), esterase (C4), leucine arylamidase and *α*-glucosidase are positive, but those for urease, *β*-glucosidase, acid phosphatase, valine arylamidase, Naphthol-AS-BI-phosphohydrolase, lipase (C14), cystine arylamidase, trypsin, *α*-chymotrypsin, *α*- and *β*-galactosidase, *β*-glucuronidase, N-acetyl-*β*-glucosamidase, *α*-mannosidase and *α*-fucosidase are negative. Cell growth ranges from 15 to 35°C and from pH 5.5 to 9.5. It is tolerant to gamma- and UV-radiation, mitomycin C, hydrogen peroxide, desiccation and heavy metals/metalloids AsO_4_^3−^, Pb^2+^, CrO_4_^2−^ and Ag^1+^. The peptidoglycan in the cell wall contains* meso*-diaminopimelic acid as diamino acid, with galactose as the diagnostic sugar. The predominant menaquinone is MK-9(H_4_). The main polar lipids are phosphatidylethanolamine, phosphatidylcholine, phosphatidylinositol, and small amount of diphosphatidylglycerol. Cellular fatty acids consist mainly of iso-C_16:0_, iso-C_15:0_, C_17:1_ *ω*8c, and C_16:1_ *ω*7c. The type strain has a genomic DNA G + C content of 74.4 mol %. The INSDC accession number for the 16S rRNA gene sequences of the type strain G18^T^ (= DSM 44209^T^ = CCUG 63018^T^) is HF970583.

## Supplementary Material

Supplementary Fig. S1: The parameter “Maximum Height” estimated from the respiration curves as measured with the OmniLog phenotyping device and discretized and visualized as heatmap using the opm package. Plates and substrates are rearranged according to their overall similarity (as depicted using the row and column dendrograms). Ochre colour indicates positive reaction; blue colour indicates negative reaction; white colour indicates ambiguous reaction. Letters (A/B) indicate each replicate of experiment.
Supplementary Fig. S2: Phenotypic dendrogram based on the parameter “Maximun Height” estimated from the respiration curves as measured with the OmniLog phenotyping device using Ward algorithm for agglomerative hierarchical clustering and correlation coefficient as a distance metric using the pvclust package. Support values approximately unbiased (AU, left) and bootstrapping (BP, right) are shown above the branches. Well supported clusters are defined by red squares.
Supplementary Fig. S3: Polar lipids profile of Geodermatophilus poikilotrophi sp. nov. G18T, after separation by two-dimensional TLC. Plate was sprayed with molydatophosphoric acid for detection of total polar lipid. DPG, diphosphadidylglycerol; PE, phosphatidylethanolamine; PC, phosphatidylcholine; PI, phosphatidylinositol; GL, unknown glycolipid. Supplementary R-code File. S4. Exemplary code to fit the model and compute the confidence intervals using the R package lethal.

## Figures and Tables

**Table 1:** Differential phenotypic characteristics of strain G18^T^ and the type strains of other *Geodermatophilus* species. Strains: 1, *G. poikilotrophi* sp. nov. G18^T^; 2, *G. obscurus* DSM 43160^T^; 3, *G. ruber* DSM 45317^T^; 4, *G. nigrescens* DSM 45408^T^; 5, *G. arenarius* DSM 45418^T^; 6, *G. siccatus* DSM 45419^T^; 7, *G. saharensis *DSM 45423^T^; 8, *G. tzadiensis* DSM 45416^T^; 9, *G. telluris* DSM 45421^T^; 10, *G. soli* DSM 45843^T^; 11, *G. terrae *DSM 45844^T^; 12, *G. africanus* DSM 45422^T^; 13, *G. normandii* DSM 45417^T^; 14, *G. taihuensis* DSM 45962^T^; 15, *G. amargosae* DSM 46136^T^. All physiological data are from this study.

Characteristics	1	2	3	4	5	6	7	8	9	10	11	12	13	14	15
Colony colour on GYM	Light-red, greenish-black	Black	Light-red, red	Light-red, black	Light-red, brown	Light-red, black	Light-red, black	Light-red, greenish-black	Black	Light red	Light red	Black	Light-red, greenish-black	Coral pink	Black
Colony surface on GYM	Moist	Dry	Moist	Moist	Moist	Moist	Moist	Moist	Dry	Moist	Moist	Dry	Moist	Moist	Dry
Utilization of															
Turanose	−	+	+	+	+	−	+	+	+	+	+	−	+	+	+
Stachyose	+	+/−	+	+/−	−	−	−	+	−	+	−	−	−	+/−	−
D-Melibiose	−	−	−	+/−	−	−	+/−	+	−	+	−	−	+/−	+	+
D-Salicin	−	+/−	−	+	+	−	+	+	+	+	+/−	−	+/−	+	−
NaCl range (w/v)															
1%	+	+	+	+	+	−	+	+	+	+/−	+	+	+	+	+/−
4%	−	+/−	+	+	−	−	+	+/−	+	−	−	+	−	+	+/−
D-Mannose	+	+	−	+	+	+	+	+	+	+	+	−	+	−	+/−
L-Rhamnose	+	−	−	+	+	−	+	+	+	+	+	−	+	−	−
Inosine	−	−	+	+/−	−	−	+	+	−	+	−	−	+	+	−
D-Sorbitol	+	+	−	−	−	+	+	+	+	+/−	+/−	−	−	+/−	+/−
D-Mannitol	+	+	−	+/−	−	+	+	+	+	+	+	−	−	+	+/−
D-Arabitol	−	+	−	+/−	−	+	+	−	+	−	−	−	−	+	−
Glycerol	+	+	−	−	+	+	−	+	+	+	+	−	+	+	−
L-Alanine	−	−	+/−	−	+	+	−	−	−	+	+	−	−	+/−	+
L-Arginine	+	−	−	−	−	+	+	+	+	−	−	−	−	−	−
L-Histidine	−	−	+	−	−	−	+	−	−	−	−	−	−	−	+/−
Pectin	+	+	−	+	−	+	+	+	+	+	+	−	+	+	−
D-Gluconic acid	+	+	−	+	−	+	+	+	+	+	+	−	+	−	−
Quinic acid	+	+	+	−	−	+	−	−	+	+	+	−	−	+	−
Predominant menaquinone(s)^a^	MK-9(H_4_)	MK-9(H_4_), MK-9(H_2_), MK-8(H_4_)	MK-9(H_4_)	MK-9(H_4_)	MK-9(H_4_), MK-8(H_4_), MK-9 (H_0_)	MK-9(H_4_), MK-8(H_4_), MK-9(H_0_)	MK-9(H_4_), MK-8(H_4_)	MK-9(H_4_), MK-9(H_0_)	MK-9(H_4_)	MK-9(H_4_), MK-9(H_0_), MK-9(H_2_)	MK-9(H_4_), MK-9(H_0_)	MK-9(H_4_)	MK-9(H_4_)	MK-9 (H_4_), MK-9(H_0_)^#^	MK-9(H_4_)
Phospholipids∗	PE, PC, PI, DPG	DPG, PC, PE, PI, PG	DPG, PE, PC, PI, 2PL, PG	DPG, PE, PC, PI, PG	PE, PC, DPG, PI, PG	PE, PC, PI, DPG, PG	DPG, PC, PI, PE, PG	DPG, PC, PE, PI, PG	DPG, PC, PE, PI, APL, PG	DPG, PME, PE, PI, 3PL^†^	DPG, PME, PE, PI, 5PL^†^	DPG, PC, PE, PI, PG	DPG, PC, PE, PI, PG	DPG, PE, PI, PIM^#^	DPG, PC, PE, PI, PG
Major fatty acids^b^	i-C_15:0_, i-C_16:0_, C_17:1_ *ω*8c	i-C_15:0_, i-C_16:0_, C_17:1_ *ω*8c	i-C_15:0_, ai-C_15:0_, i-C_16:0_, ai-C_17:0_, C_17:1_ *ω*8c	i-C_15:0_, i-C_16:0_	i-C_15:0_, i-C_16:0_	i-C_15:0_, i-C_16:0_, C_17:1_ *ω*8c	i-C_15:0_, i-C_16:0_, i-H-C_16:1_	i-C_15:0_, i-C_16:0_	i-C_15:0_, i-C_16:0_	i-C_15:0_, i-C_16:0_, C_17:0_	i-C_15:0_, i-C_16:0_, C_18:1_ *ω*9c	i-C_16:0_	i-C_15:0_, i-C_16:0_	i-C_15:0_, i-C_16:0_, C_17:1_ *ω*8c	i-C_15:0_, i-C_16:0_

+, positive reaction; −, negative reaction; +/−, ambiguous; MK, menaquinones; DPG, diphosphatidylglycerol; PE, phosphatidylethanolamine; PME, phosphatidyl-N-methylethanolamine; PE-OH, hydroxyphosphatidylethanolamine; PG, phosphatidylglycerol; PC, phosphatidylcholine; PI, phosphatidylinositol; PIM, phosphatidylinositol mannoside; PL, unknown phospholipid; APL, unknown amino-phospholipid; i-, iso-branched; ai-, anteiso-branched.

^a^Only components making up ≥ 5% peak area ratio are shown; ^b^only components making up ≥ 10% peak area ratio are shown; ∗the components are listed in decreasing order of quantity.

^†^Data taken from Jin et al. [10]. ^#^Data taken from Qu et al. [26].

**Table 2:** Minimum inhibitory concentration of seven heavy metals and metalloids for strain G18^T^ and *G. obscurus* DSM 43160^T^.

Strain	MIC (mM) of						
	AgNO_3_	CuCl_2_	CoCl_2_	NiCl_2_	K_2_CrO_4_	Pb(NO_3_)_2_	NaHAsO_4_
G18^T^	1.0	0.1	0.3	0.5	4.0	4.0	8.0
DSM 43160^T^	0.3	0.1	0.3	0.3	1.0	1.0	80.0
