# Supplementary material for: Geodermatophilus poikilotrophi sp. nov.: A Multitolerant Actinomycete Isolated from Dolomitic Marble
Source: Biomed Res Int. 2014 Jul 9;2014:914767. doi: 10.1155/2014/914767 (PMC4119925; doi:10.1155/2014/914767)
Supplement: Supplementary file 1 — Supplementary Fig. S1: The parameter “Maximum Height” estimated from the respiration curves as measured with the OmniLog phenotyping device and discretized and visualized as heatmap using the opm package. Plates and substrates are rearranged according to their overall similarity (as depicted using the row and column dendrograms). Ochre colour indicates positive reaction; blue colour indicates negative reaction; white colour indicates ambiguous reaction. Letters (A/B) indicate each replicate of experiment. Supplementary Fig. S2: Phenotypic dendrogram based on the parameter “Maximun Height” estimated from the respiration curves as measured with the OmniLog phenotyping device using Ward algorithm for agglomerative hierarchical clustering and correlation coefficient as a distance metric using the pvclust package. Support values approximately unbiased (AU, left) and bootstrapping (BP, right) are shown above the branches. Well supported clusters are defined by red squares. Supplementary Fig. S3: Polar lipids profile of Geodermatophilus poikilotrophi sp. nov. G18T, after separation by two-dimensional TLC. Plate was sprayed with molydatophosphoric acid for detection of total polar lipid. DPG, diphosphadidylglycerol; PE, phosphatidylethanolamine; PC, phosphatidylcholine; PI, phosphatidylinositol; GL, unknown glycolipid. Supplementary R-code File. S4. Exemplary code to fit the model and compute the confidence intervals using the R package lethal. [file 914767.f1.pdf]

Supplementary Fig. S1. The parameter “Maximum Height” estimated from the respiration curves as measured with the OmniLog phenotyping device and discretized and visualized as heatmap using the `opm` package. Plates and substrates are rearranged according to their overall similarity (as depicted using the row and column dendrograms). Ochre colour indicates positive reaction; blue colour indicates negative reaction; white colour indicates ambiguous reaction. Letters (A/B) indicate each replicate of experiment.

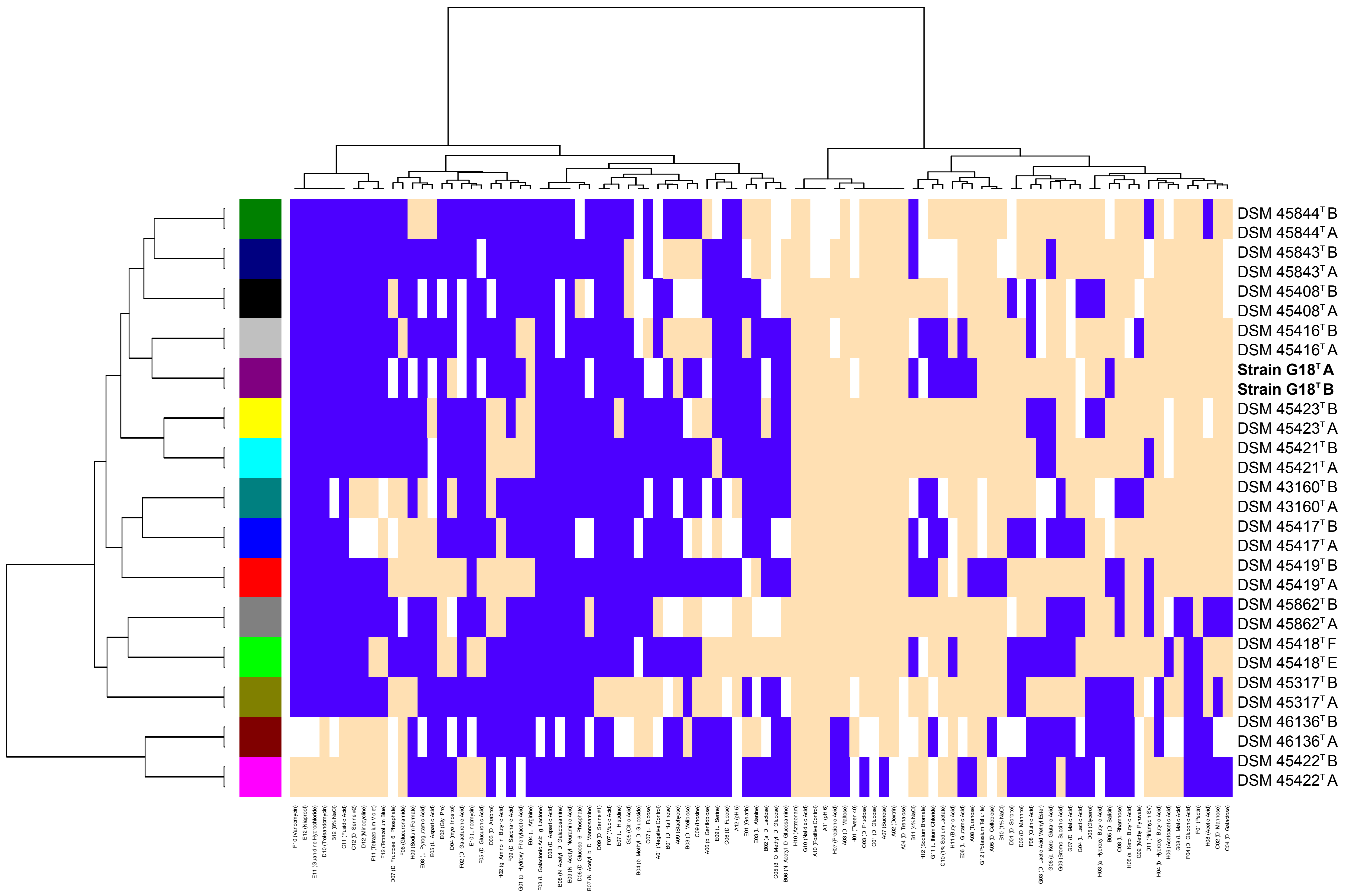
DSM 45844T B
DSM 45844T A
DSM 45843T B
DSM 45843T A
DSM 45408T B
DSM 45408T A
DSM 45416T B
DSM 45416T A
Strain G18T A
Strain G18T B
DSM 45423T B
DSM 45423T A
DSM 45421T B
DSM 45421T A
DSM 43160T B
DSM 43160T A
DSM 45417T B
DSM 45417T A
DSM 45419T B
DSM 45419T A
DSM 45862T B
DSM 45862T A
DSM 45418T F
DSM 45418T E
DSM 45317T B
DSM 45317T A
DSM 46136T B
DSM 46136T A
DSM 45422T B
DSM 45422T A

Supplementary Fig. S2. Phenotypic dendrogram based on the parameter "Maximun Height" estimated from the respiration curves as measured with the OmniLog phenotyping device using Ward algorithm for agglomerative hierarchical clustering and correlation coefficient as a distance metric using the `pvclust` package. Support values approximately unbiased (AU, left) and bootstrapping (BP, right) are shown above the branches. Well supported clusters are defined by red squares.

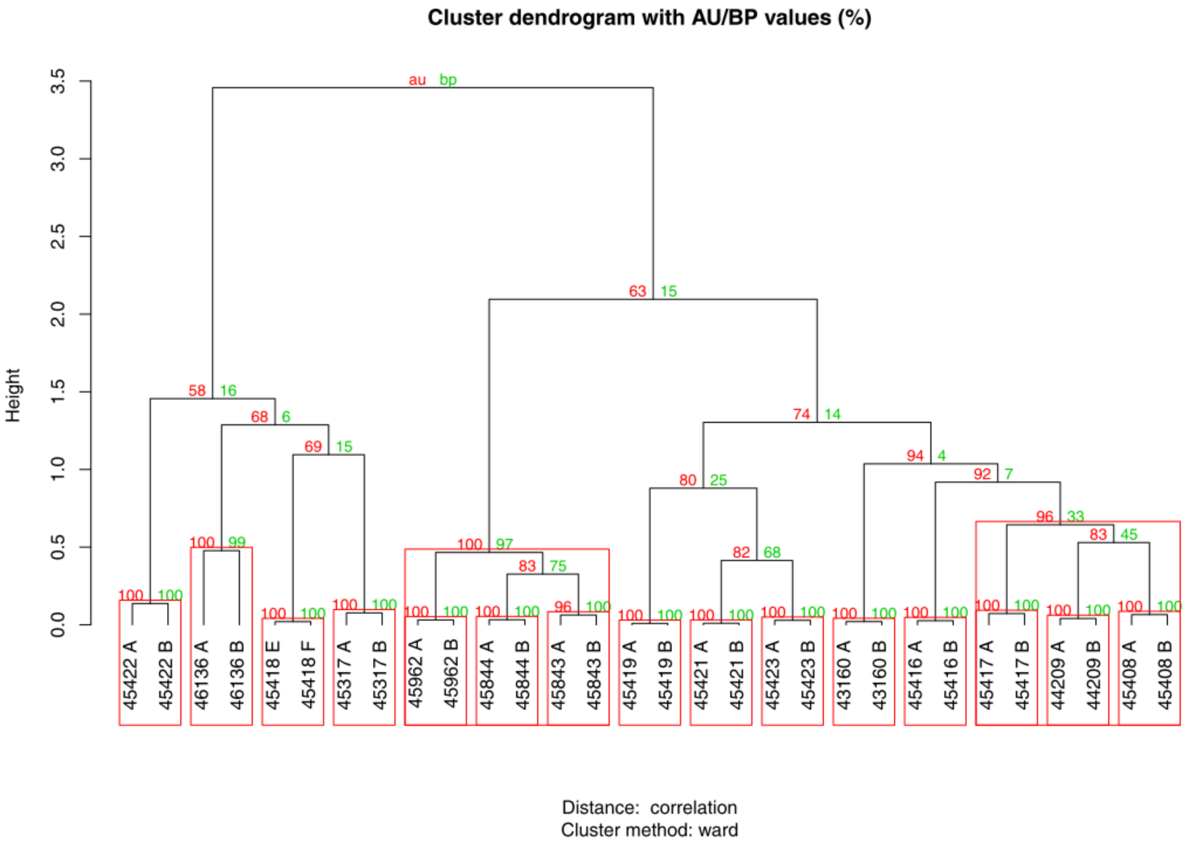

Supplementary Fig. S3. Polar lipids profile of *Geodermatophilus poikilotrophi* sp. nov. G18^{T}, after separation by two-dimensional TLC. Plate was sprayed with molydatophosphoric acid for detection of total polar lipid. DPG, diphosphadidylglycerol; PE, phosphatidylethanolamine; PC, phosphatidylcholine; PI, phosphatidylinositol; GL, unknown glycolipid.

Geodermatophilus poikilotrophi **sp. nov. G18T**

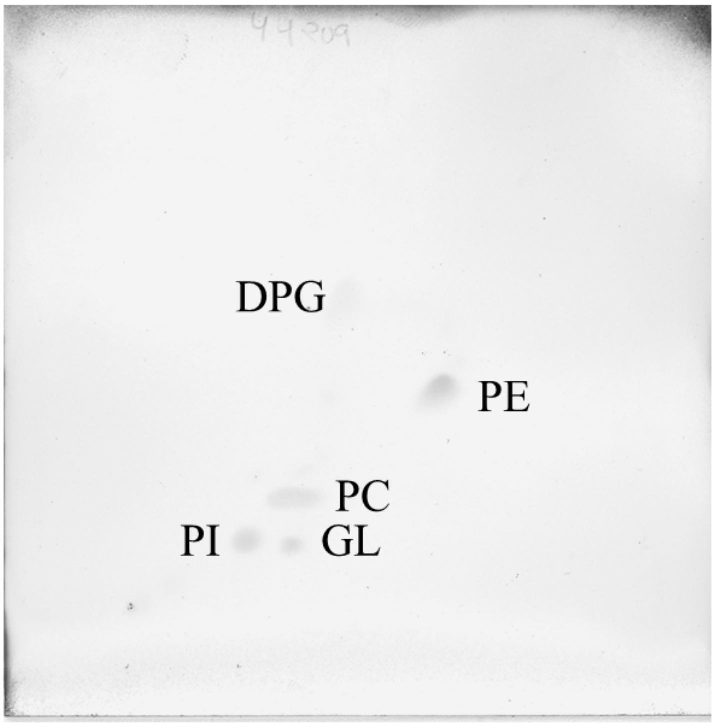

Supplementary R-code File. S4. Exemplary code to fit the model and compute the confidence intervals using the R package `lethal`.

```
################################################################################
##Extraction of lethal doses
### Example code for the manuscript
##   Geodermatophilus poikilotrophi sp. nov., a multi-tolerant actinomycete
##   isolated from dolomitic marble
```

```r
##   by Maria del Carmen Montero-Calasanz, Benjamin Hofner, Markus Göker,
##  Manfred Rohde, Cathrin Spröer, Karima Hezbri, Maher Gtari,
##  Peter Schumann,  Hans-Peter Klenk
## Author of the code: Benjamin Hofner
################################################################################
## Install and load package
install.packages("lethal", repos = "http://r-forge.r-project.org",
                 type = "source")
library("lethal")
## Run this only once as it takes some time; Thus if the results exist already,
## skip this
if (!file.exists("lethal_dose_uv.Rda")) {
    ## load UV radiation data, which is also part of the package leathal
    data("geoderm.uv")
    summary(geoderm.uv)
    ## make sure that strain and replicate are factors
    geoderm.uv$strain <- as.factor(geoderm.uv$strain)
```

```
    geoderm.uv$replicate <- as.factor(geoderm.uv$replicate)
    mod.uv <- LD(value ~ time, groups = "strain", experiment = "replicate",
                 dose_trafo = "sqrt", data = geoderm.uv,
                 family = negbin(theta = c(0.5, 10)))
    ## Set seed to make results reproducable
    ## (as we use bootstrap condidence intervals)
    set.seed(0703)
    ## On a linux machine one can easily use parallel programming by specifying
    ## the numbers of cores to use with the additional argument "mc.cores", e.g.
    ##   confint(mod.uv, B1 = 20, B2 = 100, mc.cores = 10)
    ci.uv <- confint(mod.uv)
    ## to reproduce results from the paper, one must use mc.cores = 3
    save("mod.uv", "ci.uv", file = "lethal_dose_uv.Rda")
} else {
    load("lethal_dose_uv.Rda")
}
##############################################################################
## Extract results
```

```
## extract model and LDs
mod.uv
## extract LDs only
LD(mod.uv)
## extract confidence intervals (and LDs)
ci.uv
###############################################################################
## Produce plots
## simple graphic without CIs:
plot(mod.uv, xlab = "Time (min)", ylab = expression(c.f.u.ml^-1))
## graphic with survival fractions and confidence intervals:
plot(ci.uv, xlab = "Time (min)", upper_ylab = expression(c.f.u.ml^-1),
    mar = c(4, 9.3, 2, 2.5))
## add labels
mtext(rep(c("LD10", "LD50"), 3), side = 4, at = c(0.8, 1.2, 1.8, 2.2, 2.8, 3.2),
     cex = 0.75, las = 2)
```
